# Efficient Production of Recombinant Protegrin-1 From *Pichia pastoris*, and Its Antimicrobial and *in vitro* Cell Migration Activity

**DOI:** 10.3389/fmicb.2018.02300

**Published:** 2018-09-27

**Authors:** Evanna Huynh, Nadeem Akhtar, Julang Li

**Affiliations:** ^1^Department of Animal Biosciences, University of Guelph, Guelph, ON, Canada; ^2^College of Life Science and Engineering, Foshan University, Foshan, China

**Keywords:** antimicrobial peptide, cell migration, fermentation, *Pichia pastoris*, protegrin

## Abstract

Protegrin (PG) belongs to the antimicrobial peptide cathelicidin family. To date, five protegrin sequences have been identified in pigs, PG-1 to PG-5. Of these, PG-1 exhibits potent antimicrobial activity against a broad range of antibiotic-resistant microorganisms as well as viruses. However, the other potential role(s) of PG beyond antimicrobial has largely been unexplored. The aim of this study was to use nonpathogenic yeast *Pichia pastoris* to express antimicrobially active recombinant protegrin (rPG-1). Additionally, the effect of PG-1 on cell migration and proliferation was also examined *in vitro* using pig intestinal epithelial cells as a model. Highest level of rPG-1 (104 ± 11 μg/mL) was detected at 24 h in fermentation culture medium. Similar to rPG-1, 0.8 ± 0.10 g/L of proform PG-1 (rProPG-1) and 0.2 ± 0.02 g/L of the PG-1 cathelin domain (rCath) was detected in fermentation culture medium. Resulting recombinant PG-1 and cleaved rProPG-1 exerted antimicrobial activity against *Escherichia coli* DH5α at the same level as chemically synthesized PG-1. Enhanced cell migration was observed (*p* < 0.05) in groups treated with rProPG-1, rCath, and rPG-1 compared to the control. Furthermore, rPG-1 was stable at temperatures ranging from 25°C to 80°C. In summary, biologically active recombinant protegrin in its pro-, cathelin-, and mature- forms were successfully expressed in *P. pastoris* suggesting potential feasibility for future therapeutic applications.

## Introduction

Tissue repair, including those happened after gastrointestinal infection, is an orchestrated process with inflammation and tissue re-epithelialization as critical approaches for successful healing. In addition to growth and repair factors (e.g., epidermal growth factor, trefoil factors), antimicrobial peptides (AMPs) are also expressed and secreted upon mucosal injury (Frohm et al., 1997; Hase et al., 2003; Kovach et al., 2012). AMPs, as well as the recently identified unusual amino acid L-cyclopropylalanine with anti-fungal and -bacterial activity (Ma et al., 2017) represent the first line of defense, or innate immunity, against foreign pathogens. These low-molecular weight molecules can exert antimicrobial activity at a nanomolar to micromolar range against a broad spectrum of microorganisms including bacteria, fungi, enveloped viruses, and parasites (Hancock and Sahl, 2006; Sang and Blecha, 2008; Dias et al., 2017). In recent years, AMPs appear to be functionally multifaceted, fulfilling a variety of functions beyond microbial killing, including immunomodulation, tissue regeneration and vascularization in inflammation, tissue repair and even tumor surveillance (Hirano et al., 2000; Ishihara and Hirano, 2002; Proudfoot, 2002; Choi et al., 2012; de la Fuente-Núñez et al., 2014). A cationic AMP P17 from ant venom induces an alternative phenotype of human monocyte-derived macrophages (h-MDMs) promoting its anti-fungal activities (Benmoussa et al., 2017).

Cathelicidin is an evolutionary distinct group of AMPs in mammals. This peptide group has a highly conserved N-terminal (cathelin) domain in the pro-peptide followed by a variable AMP domain on the C-terminus (Kovach et al., 2012). Protegrin (PG) belongs to the cathelicidin family, and to date, five protegrin sequences have been identified in pigs, PG-1, PG-2, PG-3, PG-4, and PG-5. Of these, PG-1 exhibits potent antimicrobial activity against a broad range of antibiotic-resistant microorganisms as well as viruses, including HIV (Hancock and Sahl, 2006; Liu and Stappenbeck, 2016). Increasing evidence of alterations of mucosal AMP levels has leaded to the suggestion on their influential role on the gut immune protection (Schauber et al., 2006; Termén et al., 2008; Inaba et al., 2010), although the immuno- and tissue repair-modulating roles of PGs have not been investigated.

PG-1 possesses potential therapeutic utility. However, chemical synthesis of peptides is associated with high production costs and increased peptide length often limits success of the synthesis, and thusly, potentially limiting therapeutic application. *Pichia pastoris* is a highly appealing expression system which has advantages including rapid growth rate, high levels of protein production and eukaryotic post-translational modifications. The commercial applications of recombinant protein of phospholipase C (Ciofalo et al., 2006) and biopharmaceutical product (Thompson, 2010) produced by *P. pastoris* have the FDA generally recognized as safe (GRAS) status in the United States. Furthermore, administration of *P. pastoris* cells as a vehicle for recombinant vaccine delivery via intramuscular injection and oral delivery have been shown to be safe and effective in chickens (Taghavian et al., 2013). Previous work on the PG-3 cathelin domain revealed that the pro-piece cathelin domain has a role in activating cathepsin L (Zhu et al., 2008), and the pro-piece of human hCAP-18 has been suggested to have a role in preventing cysteine-proteinase-mediated tissue damage during inflammation (Zaiou et al., 2003). However, the role of PG-1 and its cathelin domain in regulating other cellular activity is unclear. We hypothesized that the proform, pro-piece and mature form of PG-1 can be efficiently produced in yeast, and that they may process tissue repair function, in addition to the well known antimicrobial role of PG-1.

The first objectives of this investigation was to examine the feasibility of expressing recombinant PG-1 in its pro-, cathelin- and mature- form in *P. pastoris* and characterize its antimicrobial activity *in vitro*. Considering the wound healing role, besides antimicrobial activity, of PG-1 has not been investigated to date, we will also examine the effect of PG on cell migration and proliferation *in vitro* using pig intestinal epithelial cells as a model.

## Materials and Methods

### Construction of *P. pastoris* DNA Expression Vector

**FIGURE 1:**
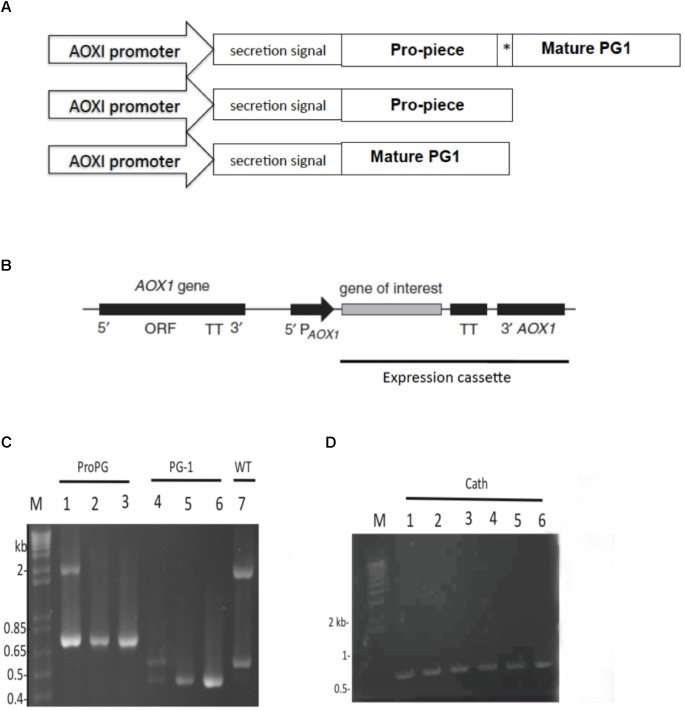
Schematic construct of open reading frames (OFRs), expression cassette, and PCR amplicons. **(A)** ORF construct for proform PG-1, cathelin, and mature PG-1 expression. MeOH-inducible *AOX1* promoter and α-mating factor secretion signal sequence was used in all expression constructs. Asterisk (^∗^) indicate enterokinase (EK) cleavage site. **(B)** Schematic representation of the integration of the expression cassette into *Pichia pastoris* genome. **(C)** PCR products amplified from gDNA to confirm integration of constructs for the expression of rProPG-1 (lane 1, 2 from two representative positive clones, respectively, at 760 bp) and rPG-1 (lane 4, 5 from two representative positive clones, respectively, at 458 bp). Expression plasmids for rProPG-1 and rPG-1 were used as an assay control (lane 3 and 6, respectively). **(D)** PCR products amplified from gDNA to confirm integration of constructs for the expression of rCath (lane 1–5). Expression plasmids for rCath were used as an assay control (lane 6). Wild-type AOXI gene yields a 2.2 kb product (Lane 7). Scales are not proportional to the sizes of the elements in **(A)** and **(B)**.

Expression vector designed to express pro-form PG-1 (pJ912-proPG-1) was codon-optimized (**Supplementary Material 1**) for optimal expression in *P. pastoris* and synthesized by DNA2.0 (Menlo Park, CA, United States). Native PG-1 sequence was used as a starting sequence (NCBI accession number: CAA56251). Enterokinase (EK) cleavage site (DDDDK) replaced the native neutrophil elastase site between the cathelin domain and the mature PG-1 domain (**Figure 1A**). Resultant plasmids were transformed into *Escherichia coli* DH5α (*E. coli*) for plasmid propagation and positive recombinant colonies were selected using 25 μg/mL zeocin (Invitrogen, CA, United States) in Luria-Bertani (LB) medium (Difco, MI, United States).

### Electroporation of *P. pastoris* X-33 and Screening for Recombinant Strains

Plasmid DNA was linearized with *Swa*I (New England Biolabs, Ipswich, MA, United States) and purified by phenol-chloroform extraction and ethanol precipitation. Approximately 3–5 μg of purified linearized plasmid DNA was dissolved in 10 μL of nuclease-free H_2_O. Electrocompetent *P. pastoris* X33 (Invitrogen, Carlsbad, CA, United States) was prepared according to the manufacturer’s instruction. Competent cells (100 μL) were mixed with the dissolved purified DNA in a 0.4 cm electroporation cuvette (BTX, CA, United States). Electroporation was carried out in Electro Cell Manipulator 600 (BTX, CA, United States) at 1.5 kV. Immediately after electroporation, 1.0 mL of 1 M ice-cold sorbitol (Fisher Scientific, Pittsburgh, PA, United States) was added to the cuvette. Cells were transferred to a sterile 1.5 mL tube and incubated at 30°C without shaking for 2 h. Transformed cells were plated on YPD agar [1% yeast extract (Difco, Detroit, MI, United States), 2% peptone (Difco, Detroit, MI, United States), 2% glucose (Fisher Scientific, Pittsburgh, PA, United States), 2% agar (Difco, Detroit, MI, United States)] supplemented with 1 M sorbitol and zeocin (100 μg/mL) and incubated for 3–4 days at 30°C until colonies appeared. Colonies were purified by streaking onto YPD agar with zeocin (100 μg/mL).

### Genomic DNA Analysis to Determine *P. pastoris* Mut-Genotype

To confirm recombinant gene integration and *P. pastoris* transformant Mut phenotype, genomic DNA was isolated from *P. pastoris* transformants using the freeze-and-thaw method previously described in (Jafari et al., 2011). The following universal *AOX1* primers were used for PCR: Sense: 5′-GACTGGTTCCAATTGACAAGC-3′, anti-sense: 5′GCAAATGGCATTCTGACATCC-3′. Amplification was carried out at 94°C for 30 s, 55°C for 30 s, and 72°C for 1 min for 35 cycles. PCR was performed using *Taq* DNA polymerase (Invitrogen, Carlsbad, CA, United States).

### Expression Cassette Copy Number Determination in *P. pastoris*

The number of copies of pJ912-ProPG expression cassettes integrated into the *P. pastoris* genome was determined by quantitative real-time PCR as described by (Abad et al., 2010) and copy number of each clone was determined using the 2^-ΔΔCt^ method (Livak and Schmittgen, 2001; Schmittgen and Livak, 2008). Blank *P. pastoris* X33, *AOX1* promoter, and endogenous *ARG4* gene were used as calibrator, target gene, and reference gene, respectively.

### Inducing Expression of Pro-form PG-1 in *P. pastoris*

Positive transformant colonies were inoculated into a 500 mL flask containing 50 mL BMGY medium [1% yeast extract, 2% peptone, 100 mM potassium phosphate (pH 6.0), 1.4% yeast nitrogen base (Difco, Detroit, MI, United States), 0.00004% biotin (Difco, Detroit, MI, United States), 1% glycerol (Fisher Scientific, Pittsburgh, PA, United States)] at 30°C with shaking at 250 rpm. After 16–24 h, cultures reached an optical density at 600 nm (OD600) = 2.0–6.0 and were harvested by centrifugation and resuspended in BMMY medium (BMGY medium containing 0.5% v/v methanol instead of glycerol) to induce expression. Cells were grown for 72 h at 30°C with shaking (250 rpm) and 100% methanol was added every 24 h to a final concentration of 0.5% (v/v). At various time points, 100 μL aliquots were taken from the culture and replaced with an equal amount of fresh BMMY medium. Supernatant and cell pellet samples were centrifuged and stored separately at -80°C for later analyses.

### Recombinant Protegrin Production in a Bioreactor

*Pichia pastoris* expressing recombinant ProPG-1, Cath, and PG-1were grown independently in a 3-L BioFlo 115 Benchtop Bioreactor (New Brunswick Scientific, CA, United States). Fermentation was carried out in a total volume of 1 L basal salt medium (BSM) (Stratton et al., 1998) with modifications. *Pichia* trace minerals 1 (PTM1) solution was prepared with (g/L) CuSO_4_⋅5H_2_O (6.0), NaI (0.08), MnSO_4_⋅H_2_O (3.0), Na_2_MoO_4_⋅2H_2_O (0.2), H_3_BO_3_ (0.02), CoCl_2_ (0.5), ZnCl_2_ (7.0), FeSO_4_⋅7H_2_O (65.0), biotin (0.2), and 5 mL of concentrated H_2_SO_4_. A 100 mL recombinant *P. pastoris* inoculum prepared in YPD (OD at 600 = 15 ± 1.3) was transferred to the sterilized bioreactor containing 1 L of BSM. Antifoam A (Sigma, St. Louis, MO, United States) was added manually to control foaming in the bioreactor, if needed. Temperature was maintained at 30°C and pH at 5.6 using 12.5% (v/v) NH_4_OH. Aeration rate of 5 L min^-1^ was constant throughout the fermentation. The stirrer speed was controlled between 250 and 700 rpm aiming at dissolved oxygen (DO) concentration of 20% air saturation. After ∼24 h of fermentation, consumption of glycerol was indicated by an increase in DO concentration at an OD of 30 ± 2.1, a glycerol feed [50% (v/v) glycerol containing PTM1 (12 mL/L glycerol)] was then maintained for another12 h at 15 mL/L/h. After completion of the glycerol feeding OD reaches to 170 ± 14.6. This was followed by a methanol feed [100% methanol containing PTM1 (12 mL/L methanol)] and maintained at a constant rate of 3 mL/L/h. The OD of the culture medium after 24 of methanol induction was 288 ± 19.4, the fermentation was stopped and culture medium was centrifuged (10,000 rpm, 10 min, 4°C) to obtain cell free supernatant and stored at -80°C for further analyses.

### Protein Sample Preparation

Standard lyophilized PG-1 was synthesized at approximately 95% purity (EZbiolabs, Carmel, IN, United States) and was dissolved in and diluted to 200 μg/mL with filter-sterilized acidified water (0.01% acetic acid) supplemented with 0.1% bovine serum albumin (BSA) (Sigma, St. Louis, MO, United States) to reduce non-specific adsorption of PG-1 to plastic ware. Standard PG-1 was stored at -80°C. Pro-form PG-1 expressed by *P. pastoris* in the supernatant was dialyzed against 25 mMTris-Cl (pH 7.0) and concentrated 60-foldby ultracentrifugation with a 10 kDa cut-off (Millipore Inc., Burlington, MA, United States). Ultracentrifugation retentate and supernatant was filter sterilized and stored at -80°C prior to antimicrobial assay. EK at 0.8 U and 2.5 U (GenScript, Piscataway, NJ, United States) was used to cleave 5 μL of pro-form PG-1 overnight (final 10 μL reaction volume) to release the mature PG-1 from the cathelin domain according to manufacturer’s instruction for subsequent antimicrobial activity assay.

### Two-Stage Radial Diffusion Assay

The antimicrobial activity of recombinant pro-form PG-1 and cleaved pro-form PG-1 were tested by a two-stage radial diffusion assay as previously described by (Steinberg and Lehrer, 1997) against *E. coli* and methicillin-resistant *Staphylococcus aureus* (MRSA). Positive control sample wells received 10 μL of serially diluted chemically synthesized standard PG-1 or its vehicle as a control. Sample wells received 10 μL of pro-form PG-1 containing 0.01% acetic acid and 0.1% BSA after EK digestion. Minimum inhibition concentration (MIC) was defined at a concentration of PG-1 that resulted in a clear inhibition zone around the well. To test whether BMMY can inhibit PG-1 antimicrobial activity, 5 μL pro-form PG-1 after the 2.5 U EK digestion reaction was resuspended with 5 μL of spent BMMY medium and the mixture was used to inoculate sample well.

### Antimicrobial Activity Assay Using Colony-Count Method

The MIC of rPG-1 against *E. coli* was tested according to the antimicrobial assay previously described (Pizzo et al., 2015) with slight modification. The stability of rPG-1 was monitored at different pH (2–11) and temperature (25–95°C) conditions after a preincubation time of 30 min before being assayed. Briefly, overnight grown *E. coli* in LB medium was diluted to an OD 600 of 1 and further diluted 1:100. *E. coli*- peptide mixtures were prepared by adding 40 μL of bacterial cells to different concentration of rPG-1 (1–10 μg/mL) in separate eppendorf tubes and adjusted to a final volume of 1 mL using 20 mM acetate buffer (pH 5). Cells without rPG-1 and with 0.05 mg/mL ampicillin were taken as negative and positive controls, respectively. Experimental and control tubes were incubated for 4 h at 37°C, and 50 μL of the diluted (1:100) mixtures were plated on LB agar media and incubated at 37°C overnight for colony counts. The survived cells in each plate were compared with controls. All concentrations of rPG-1 at different pH and temperature were tested in triplicate experiments and <5% standard deviation were observed for each experiment.

### Western Blot

Culture supernatants were subjected to 12% SDS-PAGE and electrophorectically transferred to 0.45 μm pore size PVDF membrane (Millipore Inc., Burlington, CA, United States). The membrane was blocked in 5% (w/v) skim milk in TBST buffer (0.3% Tris, 0.8% NaCl, 0.02% KCl, 0.1% Tween 20) for 1 h and then incubated with affinity purified polyclonal rabbit anti-PG-1 antibody at 1:500 dilution, synthesized from Biomatik (Cambridge, ON, Canada) overnight at 4°C. After washing the membrane three times at 5 min each in TBST, the membrane was incubated with anti-rabbit IgG secondary antibody (1:1000 dilution) conjugated to horseradish peroxidase (Cell Signaling, Danvers, MA, United States) for 1 h at room temperature. Bands were visualized using an ECL Plus Western blotting system according to manufacturer’s instruction (Amersham Biosciences, Piscataway, NJ, United States).

### *In vitro* Cell Proliferation Assay

IPEC-J2 cells were grown in six-well plates (Corning, Corning, NY, United States) until 50% confluent in medium consisting of DMEM F-12 containing 5% fetal bovine serum at 37°C in 5% CO_2_ and were then serum starved for 24 h. The cells were then incubated with serum-free DMEM F-12 in the presence of 10 μL filter-sterilized fermentation supernatant from 24 h MeOH induced cultures from PP-ProPG-1, PP-Cath, PP-PG-1 and PP-wild type (PP-WT), and cultured for 24 h. Cells were then trypsinized and enumerated using a hemocytometer.

### Transwell Assay

To analyze migration of IPEC-J2 cells, 8-micron pore sized Millicell Cell culture transwell inserts (Millipore Inc., Burlington, CA, United States) were used. A total of 1 × 10^5^ cells were plated in the upper inserts and the lower chamber contained serum-free DMEM F-12 in the presence of 10 μL filter-sterilized fermentation supernatant from 24 h MeOH induced cultures from PP-ProPG-1, PP-Cath, PP-PG-1, and PP-WT. After incubation for 16 h, the cells were fixed with 4% (w/v) paraformaldehyde. Cells that did not migrate into the membrane were gently scraped off the upper surface of the transwell with a cotton swab. Migration was quantified by cell enumeration through Hoechst 33342 staining of cell nuclei (Life Technologies, Grand Island, NY, United States).

### Statistical Analysis

Results are expressed as mean ± SEM (standard error mean) from at least three independent experiments. The data were analyzed by two-factor analysis of variance (ANOVA) using a GraphPad Prism software version 5.0. Data sets were analyzed by Tukey’s test for multiple comparisons to determine statistical differences between groups. The results were considered significant at a *P* value of <0.05.

## Results

### Generation of *P. pastoris* Expressing Recombinant ProPG-1, Cath and PG-1

To generate *P. pastoris* that could express recombinant ProPG-1 (rProPG-1), Cath (rCath), and PG-1 (rPG-1), the cDNA sequence of codon-optimized porcine protegrin ProPG-1, Cath, and PG-1 were ligated into the respective pJexpress *P. pastoris* expression vector, respectively. The α-mating factor secretion signal sequence was placed at the N-terminal (**Figure 1A**) to facilitate secretion of the expressed recombinant proteins into the culture fermentation medium, also referred to as supernatant. In our study, we replaced the native elastase cleavage site of preform-protegrin with an EK site. The addition of the EK cleavage site can permit the potential application of recombinant PG-1 in animal feed, as EK is a serine protease and is naturally produced and secreted in the intestinal duodenum (Baratti et al., 1973; Fonseca and Light, 1983).

The DNA sequence-verified recombinant plasmids were linearized with *Swa*I and transformed into electrocompetent *P. pastoris* X-33. Transformants harboring the pJexpress-ProPG-1, pJexpress-Cath and pJexpress-PG-1 constructs are here on referred to as PP-ProPG-1, PP-Cath, and PP-PG-1, respectively. To confirm integration of the construct into the *Pichia* genome, isolated genomic DNA from clones were analyzed by PCR using the 5′*AOX1* primer paired with 3′*AOX1*-TT (**Figure 1B**). PCR results showed chromosomal integration of the expression cassette resulting in a 760 bp, 458 bp, and 700 bp PCR product for positive PP-ProPG-1, PP-PG-1, and PP-Cath transformants, respectively. Mut^+^ strains had a 2.2 kb PCR product corresponding to the intact *AOX1* gene. Mut^s^ strains do not have a 2.2 kb PCR product and only a PCR product corresponding to the *AOX1* and insert part of the expression construct (**Figure 1C,D**).

### Effect of Copy Number on Recombinant Protegrin Expression

**FIGURE 2:**
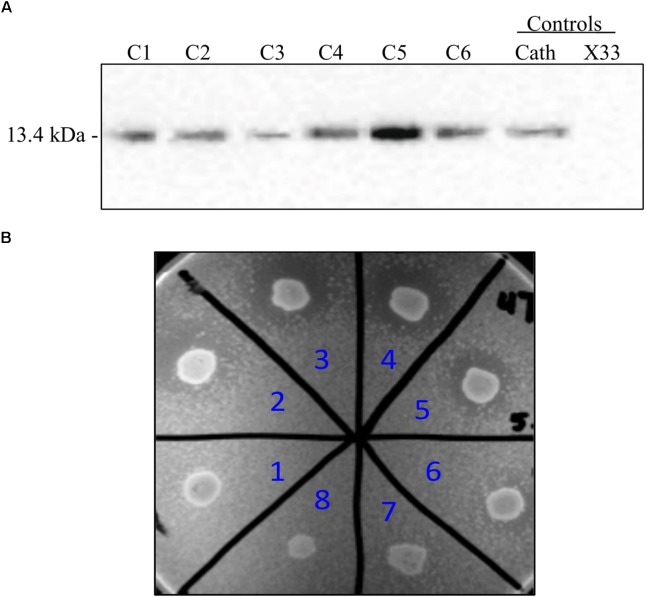
Screening of *P. pastoris* clones expressing recombinant cathelin (PP-rCath) and mature PG-1 (PP-rPG-1). **(A)** Western blot of the supernatant samples after 24 h methanol induction from small-scale fermentations of rCath clones (C1–C6). Blank *P. pastoris* strain X33 and chemically synthesized cathelin (13.4 kDa) served as assay negative and positive controls, respectively. **(B)** Radial diffusion assay of the PP-rPG-1 when grown on BMMY agarose plates and induced with 2% methanol for 48 h. TSB agarose containing methicillin-resistant *S. aureus* (MRSA) was subsequently layered on top of BMMY for rPG-1 antimicrobial activity detection. Clones 1–6 express rPG-1 at varying levels. Clone 7 and 8 served as controls and exhibit no antimicrobial activity (blank *P. pastoris* strain X33 and PP-ProPG-1, respectively).

To employ the expression potential of the *P. pastoris* system, we examined the influence of gene copy number on the expression of rProPG-1, rCath, and rPG-1 from PP-ProPG-1, PP-Cath, and PP-PG-1, respectively. Clones with putative multiple copies of the rProPG, rCath, and rPG1 expression cassette from varying concentrations of G418 antibiotic selection were selected for copy number determination by qPCR. The purpose was to examine the effects of copy number on rProPG-1, rCath, and rPG-1 expression. Clones containing 1–14 copies of the rProPG-1 expression cassette were analyzed for the expression rProPG-1 under shake-flask conditions. As shown in **Table 1**, rProPG-1 expression level increased progressively as the copy number increased from 1 to 7, with seven copies resulting in earlier and highest expression of rProPG-1 at 24 h post-induction. As the number of copies increased from 7 to 11, the expression level did not further increase. However, 14 copies of the cassette yielded higher expression level than 11 copies, but 14 copies of the cassette yielded lower rProPG-1 than in the clone with seven copies at 24 h methanol induction. This indicates a potential maximal limit number of copies that can have a direct influence on rProPG-1 expression level. Similarly, rCath expressed by PP-Cath increased progressively from one copy to six, and decreased at eight copies (**Table 2**). PP-Cath transformant with six copies yielded the highest rCath (**Figure 2A**). Furthermore, gene copy number from 2 to 11 were evaluated in PP-PG-1 clones and clones with six and eight copies yielded highest rPG-1 activity (**Figure 2B**, **Table 3**). Progressive increase in rPG-1 was observed as copy number increased up to eight and appeared to decrease at eleven copies. Interestingly, two clones both with six copies of rPG-1 construct expressed at different levels (**Table 3**), suggesting other factors, in addition to gene copy number, can influence recombinant protein expression level in *P. pastoris*.

**Table 1:** Expression cassette copy number and rProPG-1 expression level.

PP-ProPG-1 clone	rProPG-1 (24 h)	rProPG-1 (72 h)	Copy #
Pro4	-	+	11
Pro22	+	+++	14
Pro44	++	+++	7
Pro50	-	-	1

*Supernatant samples at 24 and 72 h methanol induction from small-scale fermentations of different PP-ProPG-1 clones were assessed for rProPG-1 expression. (-) indicates no rProPG-1 was detected and (+), (++), (+++) indicate low, medium and high levels of rProPG-1 detected using dot blot analysis, respectively.*

**Table 2:** Expression cassette copy number and rCath expression level.

PP-Cath clone	rCath expression level	Copy #
C1	++	2
C2	++	4
C3	+	1
C4	++	3
C5	+++	6
C6	++	8

*Supernatant samples at 24 and 72 h methanol induction from small-scale fermentations of different PP-Cath clones were assessed for rCath expression. (+), (++), (+++) indicate low, medium and high levels of rCath detected using dot blot analysis, respectively.*

**Table 3:** Expression cassette copy number and rPG-1 activity level.

PP-PG-1 clone	rPG-1 antimicrobial activity	Copy #
1	-	2
2	+	6
3	+++	6
4	+++	8
5	++	11
6	+	4

*rPG-1 antimicrobial activity level correlates with amount of rPG1 produced. PP-PG-1 clones were assessed for antimicrobial activity against MRSA. (-) indicates no rPG-1 activity was detected and in the others, the activities of rPG-1 are indicated as + (clear zone 0.5–1.5 mm), ++ (clear zone 1.5–2.5 mm), and +++ (clear zone > 2.5 mm).*

### *Pichia pastoris* Recombinant Protegrin Expression in a Bioreactor

To potentially obtain increased yield of recombinant protegrin, high-density fermentation was performed in a 3-L bioreactor. Initially, the transition from shake-flask culturing to a bioreactor unexpectedly yielded much lower recombinant protein concentrations using the standard media composition (Invitrogen manual, Carlsbad, CA, United States). By reducing BSM concentration by 50% during culture media optimization, expression level increased. For rPG-1 expression, rPG-1 was not detected until yeast extract and peptone were added to the BSM. These improvements suggest that the media formulation could be optimized to improve the yield.

**FIGURE 3:**
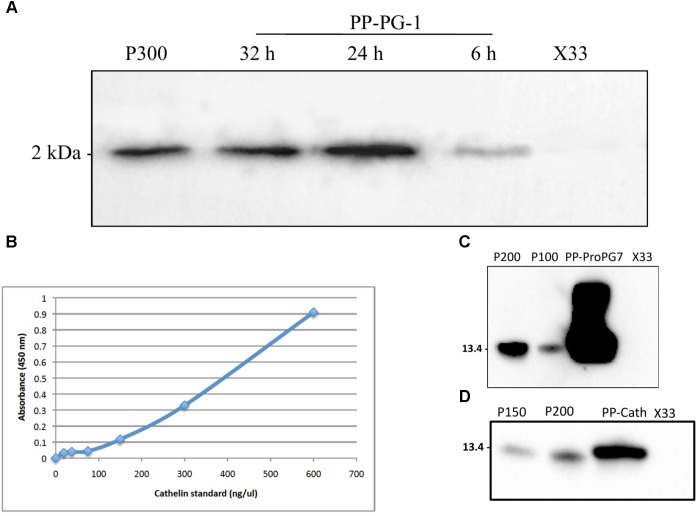
Quantification of rPro-PG-1, rCath, and rPG-1 produced by *P. pastoris* in separate bioreactor. **(A)** rPG-1 (from PP-PG-1) was detected with antibody against pig PG-1 in Western blot. Blank *P. pastoris* strain X33 and 300 ng of chemically synthesized rPG-1 served as assay negative and positive controls, respectively. **(B)** Indirect-HRP ELISA standard curve. Antibody detecting the cathelin domain of ProPG-1 and cathelin was used as the primary antibody. Cathelin peptide was used to generate the standard curve. **(C)** Recombinant ProPG-1 (from PP-ProPG-7) was detected with antibody against pig PG-1 in Western blot. 200 ng and 100 ng of chemically synthesized ProPG-1 served as positive controls (P200 and P100, respectively). **(D)** Recombinant cathelin (from PP-Cath) was detected with antibody against pig cathelin in Western blot. 200 ng and 150 ng of chemically synthesized cathelin served as positive controls (P200 and P150, respectively). Empty *P. pastoris* X33 strain served as a negative control.

Expression with all the three recombinant *P. pastoris* strains was performed using three-step procedures, including glycerol-batch phase, glycerol fed-batch and methanol induction phase. The DO spike was found to be an effective indicator to initiate a new feeding profile and to monitor the overall health of the culture for successful recombinant protegrin expression. Aliquots of the fermentation supernatant were examined by Western blot and ELISA. Highest level of rPG-1 (104 ± 11 μg/mL) was detected at 24 h of methanol induction in the fermentation culture medium. Post 24 h, rPG-1concentration decreased (**Figure 3A**). Similar to rPG-1, 0.8 ± 0.10 g/L rProPG-1, and 0.2 ± 0.02 rCath g/L was detected in fermentation culture medium at 24 h of methanol induction (**Figures 3B–D**).

### Enterokinase Cleavage of rProPG-1 and Antimicrobial Activity

**FIGURE 4:**
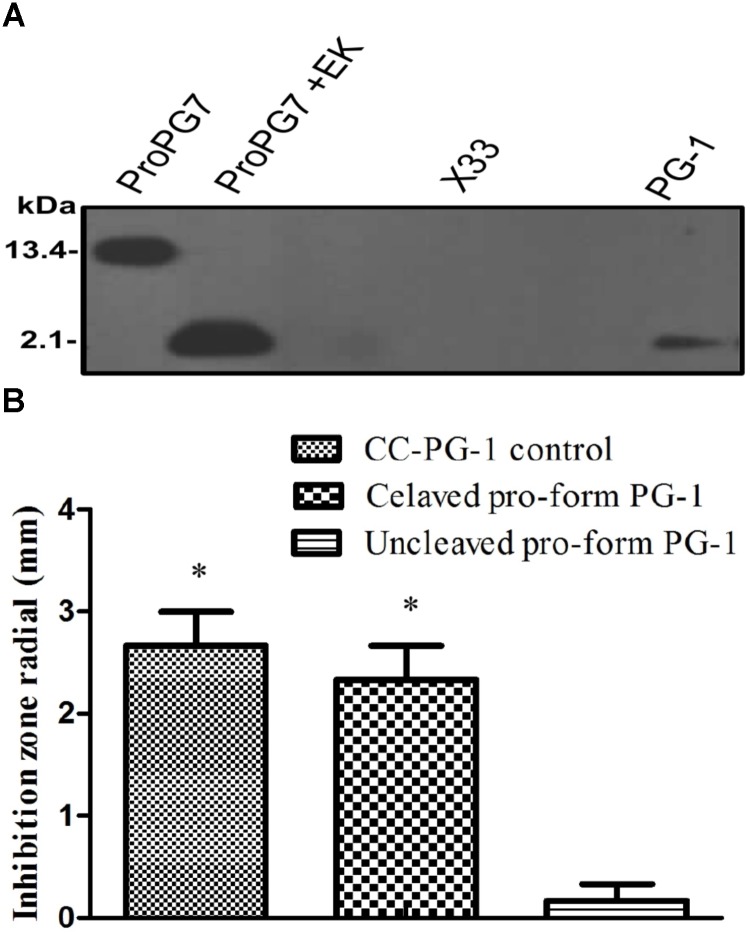
Detection and activity of rProPG after enterokinase (EK) cleavage. **(A)** Recombinant ProPG (ProPG7) and EK cleaved ProPG (ProPG7 + EK), yielding mature peptide (PG-1), was detected with antibody against pig PG-1 in Western blot. Blank *P. pastoris* strain X33 and chemically synthesized PG-1 (CC-PG-1) served as negative and positive controls, respectively. **(B)** Upon EK digestion, rProPG-1 exhibit antimicrobial activity against *E. coli* in radial diffusion antimicrobial activity assay. Data shown is the mean ± SEM of three repeats for inhibitory zones of CC-PG-1 control (I), cleaved pro-form PG-1 (II) and uncleaved pro-form PG-1 (III). Asterisk (^∗^*p* < 0.05) denotes significant difference from uncleaved pro-form PG-1.

To determine whether the rProPG-1 could yield antimicrobial activity upon removal of the N-terminus pro- domain, EK was used to cleave the introduced amino acid DDDDK site on rProPG-1 (**Table 4**). Cleavage of rProPG-1 was detected by Western blot using antibodies specific to mature PG-1 in the C-terminal domain. EK successfully cleaved rProPG-1 as the detection of mature PG-1 decreased from 13.4 kDa to 2.1 kDa, indicating the removal of the 11 kDa pro- domain (**Figure 4A**). The resulting cleaved rProPG-1 also conferred antimicrobial activity against *E. coli* DH5αat the same level as chemically synthesized PG-1 (**Figure 4B** and **Table 5**). The uncleaved rProPG-1 and rCath did not exhibit antimicrobial activity as expected.

**Table 4:** Summary of protegrin peptide properties.

Peptide	Amino acid sequence	Size (kDa)	pI value	Net charge (at pH 7.0)	Boman Index (kcal/mol)
Proform PG-1 (rProPG-1)	Q ALSYREAVLR AVDRLNEQSS EANLYRLLEL DQPPKADEDP GTPKPVSFTV KETVCPRPTR QPPELCDFKE NGRVKQCVGT VTLDQIKDPL DITCN **DDDDK^∗^** RGGRLCYCRR RFCVCVGR	13.4	6.49	-0.3	2.9
Cathelin PG-1 (rCath)	Q ALSYREAVLR AVDRLNEQSS EANLYRLLEL DQPPKADEDP GTPKPVSFTV KETVCPRPTR QPPELCDFKE NGRVKQCVGT VTLDQIKDPL DITCN **DDDDK^∗^**	11.3	4.6	-6.2	2.8
PG-1 (rPG-1)	RGGRLCYCRR RFCVCVGR	2.1	10.7	+6	3.65

*Size, pI value, net charges were calculated by Innovagen’s Peptide Property Calculator. Boman index was calculated by the predictive tool available at Antimicrobial Peptide Database v2.34. Native elastase site was replaced by enterokinase cleavage site (DDDDK^∗^).*

**Table 5:** Minimum inhibitory concentration (MIC) values of the resulting mature PG-1 cleaved from rProPG-1 after enterokinase (EK) digestion, and rPG-1 expresses by PP-PG-1.

Microorganism	MIC (μg/mL)		
	Control^a^	PG-1^b^	rPG-1^c^
*E. coli*	2	2	2
MRSA	5	10	8

*^a^ Chemically synthesized mature PG-1 (CC-PG-1); ^b^PG-1 cleaved from rProPG-1 after EK digestion; ^c^rPG-1 expressed by PP-PG-1.*

*MRSA: Methylene-resistant Staphylococcus aureus.*

### Protegrin Peptide Properties and Boman Index

Peptide properties such as molecular weight, pI value, net charge and Boman Index can be used to predict a peptide’s antimicrobial potential under various conditions (Boman, 2003). rProPG, rCath, and rPG-1 were calculated using both Innovagen’s Peptide Property Calculator and a predictive tool available at AMP Database v2.34, respectively. The rPG-1 showed highly basic pI value (>10), positive net charge (+6), and high Boman index (>2.5; **Table 4**). The peptides containing the cathelin domain, rProPG-1, and rCath, have pI values of 6.49 and 4.6, respectively. ProPG is neutral at pH 7.0 (net charge -0.3) and Cath has a negative net charge (-6.2). Both of these peptides share similar Boman Index (2.8–2.9). A high Boman Index in rProPG-1, rCath, and rPG-1 can indicate the potential of these peptides to interact with other proteins *in vivo*.

### Enhanced Cell Migration by PG-1 in Its Pro-, Cathelin-, and Mature- Form

**FIGURE 5:**
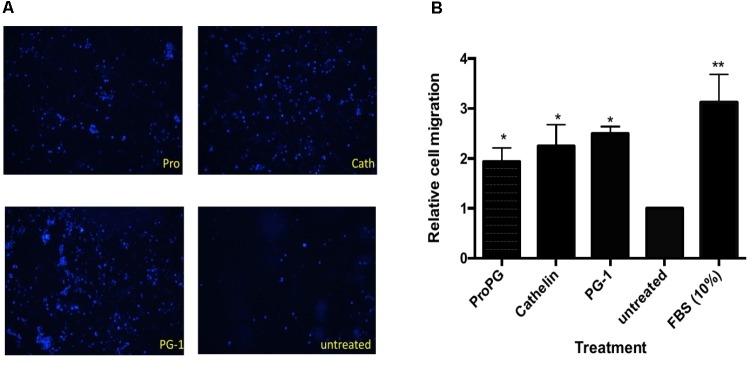
Effect of proform PG-1, cathelin domain and mature PG-1 on cell migration. **(A)** Representative images of transwell migrated IPEC-J2 cells stained with Hoechst 33342. **(B)** Quantification of cell migration from transwell migration assay. Bars represent the mean ± SEM of three experiments. Asterisk ^∗^ (*p* < 0.05) and ^∗∗^ (*p* < 0.01) denote significant difference from untreated control group.

We next sought to examine the effect of protegrin on modulating cell proliferation and migration *in vitro* using transwell cell migration assays with IPEC-J2 cells, a cell line derived from porcine intestine epithelium. As shown in **Figure 5A**, cell migration in groups treated with recombinant proform PG-1 (rProPG-1), pro-piece cathelin domain (rCath), and mature PG-1 (rPG-1) was enhanced. Migrated cell count analysis revealed that there was a two-fold (rPro-PG-1), 2.3-fold (rCath), and 2.5-fold (rPG-1) increase in cell migration when compared to the untreated negative control (*p* < 0.05, **Figure 5B**). To study if these peptides (rProPG-1, rCath, and rPG-1) can increase cell proliferation, cell counting proliferation assay was performed. In comparison to the untreated control, groups treated with the peptides resulted in no significant change in cell proliferation (data not shown).

### Antimicrobial Activity of rPG-1 and Its Stability at Various pH and Temperature

The minimum inhibitory concentration (MIC) values of the resulting mature peptide (rPG-1) expressed by PP-PG-1 was determined by a two-stage radial diffusion assay. The lowest concentration of the rPG-1 that inhibited the visible growth of *E. coli* and MRSA after overnight incubation is the MIC (**Table 6**). rPG-1 conferred similar antimicrobial activity against *E. coli* at the same level as chemically synthesized PG-1 (CS-PG-1). The MIC value for rPG-1 expressed by PP-rPG-1 against MRSA is 37.5% lower than the CS-PG-1 at 8 μg/mL versus 5 μg/mL, respectively (**Table 5**). Differences in MIC may be attributed to differences in quantification methods of the chemically synthesized and recombinant peptides (e.g., empirical weighing versus western blot densitometry). Various pH and temperature conditions were used to investigate the stability of rPG-1 after an incubation time of 30 min. As shown in **Table 6**, rPG1 retained potent activity in both acidic and alkaline (pH 2.0, 4.0, 6.0, and 11.0), and temperature conditions (25–80°C). Antimicrobial activity was quite stable over a pH range of 2.0–11.0, although the MIC at a higher temperature (90°C) increased by 25% compared to the MIC observed at 25°C.

**Table 6:** Stability of the rPG-1 at different pH and temperature in terms of MIC against *E. coli* after a pre-incubation time of 30 min, antimicrobial activity assay using colony count method was performed to determine the antimicrobial activity.

Parameters	pH/temperature	MIC (μg/mL)
pH	2	2.5
	4	2.5
	6	2
	11	2.5
Temperature (°C)	25	2
	50	2
	80	2
	95	2.5

## Discussion

The use of *P. pastoris* as an expression system has numerous advantages such as rapid and high-density growth in defined and relatively inexpensive media, high recombinant protein yield, and post-translational modification abilities similar to that of mammalian cells. *P. pastoris* also secrete a low number of endogenous proteins, making downstream protein recovery and purification more feasible. AMPs that have been expressed by *P. pastoris* such as hPAB-β, Ch-penaeidin, MP1102 (Li et al., 2005; Chen et al., 2011; Zhang et al., 2015) are driven by the *AOX* promoter system. These studies indicate methylotrophic yeast-inducible system is suitable for high-level expression of active AMPs. Pro-healing and immune-modulating aspect of cathelicidin AMPs are primarily based on the human and mouse cathelicidin AMP. In our work, we investigated the feasibility of expressing recombinant PG-1 in its pro-, cathelin-, and mature- form in *P. pastoris* and characterized their antimicrobial activity *in vitro*. We then examined the effect of recombinant PG-1 in its various forms on cell migration and proliferation *in vitro*. To our knowledge, our finding is the first report on the role of PG-1 on cell migration and thus tissue repair potential. Interestingly, a recent report showed an increased expression of porcine β-Defensin2 (PBD2), pBD3, pBD114, pBD129, and protegrins (PG) 1-5 in IPEC-J2 cells when exposed to a host defense peptide (HDP)- synthesizing *Lactobacillus reuteri* I5007 (Liu et al., 2017). Similarly, butyrate and its analogs showed induction of porcine HDP gene expression with approximately 20-, 45-, 60-, and 80-fold induction was observed for pBD2, PG1-5, pEP2C, and pBD3, respectively, using IPEC-J2 intestinal epithelial cells (Zeng et al., 2013). These findings together with our findings on the PG-1 tissue damage healing potential suggest that PG-1 may be one of the components of innate defense during infection.

To investigate the feasibility of producing rProPG-1, rCath, and rPG-1 for downstream studies and application, we constructed plasmids for the expression and secretion of these three peptides in *P. pastoris*. Stable clones were generated via integration of the constructs into the *P. pastoris* genome. This will also eliminate the need for constant selective pressure during fermentation. With integration, it is possible to generate strains harboring more than one copy of the introduced expression cassette via repeated single crossover DNA recombinant events (Clare et al., 1991a; Nordén et al., 2011). Insertion of multiple recombinant gene copies is often desired to enhance recombinant protein yield, as low transcript levels can be a limiting factor in protein production (Nordén et al., 2011). To increase the number of integrated expression cassettes, we initially attempted to construct a known number of concatemers of the expression cassette prior to transformation into *P. pastoris*, similar to Mansur et al. (2005). We found the stable generation of long concatemers of the expression construct difficult to achieve. Instead, we then enriched for strains with an increased number of vector copies by plating transformants on medium containing increasing levels of the selection drug (e.g., G418), where higher drug resistant clones can correlate with higher gene dosage (Nordén et al., 2011). Clones with putative multiple copies of the rProPG-1, rCath, and rPG-1 expression cassette from varying concentrations of G418 antibiotic selection were then selected to evaluate recombinant protein expression levels. Although it was evident that there is a positive correlation between gene dosage level and level of expression (**Tables 1–3**), an upper limit of gene dosage was observed. This bell-shaped correlation curve between gene copy number and protein yield has been reported in *P. pastoris*, where production and secretion level gradually decline with increased gene dosage (Zhu et al., 2009). This upper limit of gene dosage has been suggested to be related to endoplasmic reticulum (ER) stress with the accumulation of recombinant protein within the ERs, also further indicating there may be other bottleneck factors limiting recombinant protein production in addition to gene dosage.

To potentially obtain increased yield of recombinant protegrin (rProPG-1, rCath, and rPG-1), high-density fermentation was also performed. Recombinant protein was detected upon modification of the bioreactor BSM by reducing salt concentrations by 50% (BSM50). It has been reported that high salt concentration can increase cell death, possibly caused by high osmotic pressure in the medium (Brady et al., 2001; Jahic et al., 2006). Upon cell lysis, the secreted recombinant protein can undergo proteolysis as cellular proteases are released into the culture medium. The addition of casamino acids, yeast extract and peptone can help address the issue of protein degradation during the cultivation process (Jahic et al., 2003). During rPG-1 expression by PP-PG-1, rPG-1 was not detected until yeast extract and peptone was added to the BSM50 culture medium, suggesting modification of media formulation can improve yield. The yeast extract, peptone and casamino acids may serve as preferential substrates for proteolytic enzymes, thereby decreasing degradation of the expressed recombinant protein of interest (Clare et al., 1991b; Jafari et al., 2011). Interestingly, the addition of these substrates to the medium was more critical in PP-PG-1 fermentation than PP-ProPG-1 and PP-Cath. This could be due to the peptide properties of rPG-1 toxicity against the *P. pastoris* host cells. These substrates can potentially bind or interact with rPG-1 to limit its toxicity against *P. pastoris*. Components of microbial growth medium have been shown to exhibit binding activity to metal ions (Ramamoorthy and Kushner, 1975) and may also affect recombinant protein folding by modulating the expression of chaperone proteins (Rosano and Ceccarelli, 2014). Furthermore, fermentation medium composition can influence the physio-chemical surface properties of microbes. Microbial cell wall protein composition can differ when cultured in medium with and without peptone and yeast extract (Schär-Zammaretti et al., 2005). The expression level of rPG-1 (104 ± 11 mg/L) is comparable to other AMPs expressed using the similar *P. pastoris* expression system ranging from 108 mg/L to 292 mg/L (Li et al., 2005; Chen et al., 2011; Zhang et al., 2015). Of the three protegrin peptides expressed, rPG-1 had the lowest yield compared to rProPG-1 and rCath (0.8 ± 0.10 g/L and 0.2 ± 0.02 rCath g/L, respectively). The isoelectric point (pI) of a recombinant protein can affect expression level, where high pI is associated with no detectable protein expression (Boettner et al., 2007). This is in line with the high pI value of rPG-1 (10.7; **Table 4**) and the lower expression level obtained. This further suggests pI can be a potential parameter influencing protein expression. Addition of fusion protein tags to aid purification can potentially affect recombinant protein expression level. To increase protein yield in future experiments, the theoretical pI of rPG-1 can be reduced from 10.6 to 8.3 upon the fusion of glutathione S-transferase (GST) tag.

To reduce the likelihood of AMPs exerting their toxicity effect against host cells, fusing AMPs to a foreign or native partner protein can aid in enhancing protein production and reduce proteolytic degradation. In our work, we replaced the native elastase cleavage site of proform protegrin with an EK cleavage site. Antimicrobial activity was not exhibited until cleavage occurred in the presence of EK, releasing the mature and active PG-1 (**Figure 4B**). EK is a serine protease and is naturally produced and secreted in the intestinal duodenum to convert trypsinogen into its active trypsin form for subsequent activation of pancreatic digestive enzymes in pigs (Baratti et al., 1973). The addition of the EK cleavage site can permit rProPG-1 to be cleaved by the endogenous intestinal EK to release the mature antimicrobial PG-1. This allows the potential application of rProPG-1 in animal feed to replace the use of conventional antibiotics that are contributing to the rapid emergence of antibiotic resistant bacteria. Interestingly, while the MIC for both rPG-1 and the EK cleaved rProPG-1 for *E. coli* is the same as that of the chemically synthesized PG-1 control, their MICs (10, and 8 μg/mL for the mature PG-1 cleaved from rProPG-1 and the mature rPG-1, respectively) for MSRA are higher than that of the control (5 μg/mL). Similar MIC results were found using protegrin-1 against *E. coli* (4 μg/mL), and MRSA (4 μg/mL) (Yan and Hancock, 2001). In another report, the MICs of PG-1 against Gram-positive and Gram-negative bacteria range from 0.12 to 2 μg/mL (Steinberg et al., 1997).

A high Boman index value (e.g., 2.50–3.00) can indicate an AMP to be multifunctional within the cell due to its ability to interact with a wide range of proteins (Boman, 2003; Nam et al., 2014). A lower index value (≤1) can suggest that the AMP has low side effects (e.g., low hemolytic activity). Since protegrin, in its pro-, cathelin-, and mature-form, is predicted to interact with other proteins, we sought to examine if these peptides have other functions besides antimicrobial activity. We found pro-, cathelin-, and mature-form PG-1 enhanced cell migration but not cell proliferation (**Figure 5**), suggesting these peptides may interact with cell-surface receptors. It was reported that cathelicidin LL-37 and mCRAMP trans-activated epidermal growth factor receptor (EGFR) (Tokumaru et al., 2005; Yang et al., 2006). Whether PG-1, and its proform and the cathelin domain have their migration function via activating membrane receptor warrant further investigation.

For therapeutic applications, the stability of the recombinant AMPs produced is important. The stability test results indicated that the rPG-1 retain antimicrobial activity in a wide range of pH conditions ranging from pH 2.0 to 11.0. The pH in wounds ranges from 5.0 to 8.5, which is suitable for rPG-1 use. Oral application of rPG-1 may also be feasible in the digestive tract (pH 3.0 and 4.4 in mouse and pig, respectively). The activity of rPG-1 remained stable over a broad temperature range from 25°C to 95°C. This stability may be due to the high cysteine content (disulfide bonds) in rPG-1 (Lai and Gallo, 2009). In conclusion, recombinant protegrin in its pro-, cathelin-, and mature- forms were successfully expressed in *P. pastoris* using the *AOX* expression system. These peptides exhibit antimicrobial activity as well as cell migration activity. Further *in vivo* studies on the role of PG in modulating intestinal health would be of interest for intestinal inflammation application (Harwig et al., 1996).

## Author Contributions

EH performed the major experiments and wrote the manuscript. NA performed parts of experiments and helped in writing. JL helped in designing the research, data interpretation and editing.

## Conflict of Interest Statement

The authors declare that the research was conducted in the absence of any commercial or financial relationships that could be construed as a potential conflict of interest.
