# Supplementary material for: Efficient Production of Recombinant Protegrin-1 From Pichia pastoris, and Its Antimicrobial and in vitro Cell Migration Activity
Source: Front Microbiol. 2018 Sep 27;9:2300. doi: 10.3389/fmicb.2018.02300 (PMC6170612; doi:10.3389/fmicb.2018.02300)
Supplement: Supplementary file 1 [file Data_Sheet_1.DOCX]

**Supplementary material-1**

Efficient production of recombinant protegrin-1 from Pichia pastoris, and its antimicrobial and in vitro cell migration activity

Evanna Huynh^1^, Nadeem Akhtar^1^, Julang Li^1,2,*^

^1^Department of Animal Biosciences, University of Guelph, Ontario, N1G 2W1, Canada

^2^College of Life Science and Engineering, Foshan University, Foshan, Guangdong province China

Codon-optimized sequences of the protegrin-1(PG-1) are as follows:

Mature PG-1 ORF:
AGAGGTGGAAGATTGTGCTACTGTAGGCGAAGATTCTGTGTTTGCGTAGGAAGGTAA

Pro-PG-1 ORF:
CAAGCTCTGTCCTATCGTGAGGCAGTGTTAAGAGCTGTGGACCGTCTTAACGAGCAATCATCTGAGGCTAACTTGTACAGACTTTTGGAACTGGATCAGCCCCCAAAGGCAGACGAAGATCCTGGCACCCCTAAACCAGTTAGCTTTACAGTGAAGGAAACAGTCTGTCCACGTCCGACGAGACAACCTCCAGAGCTATGTGATTTCAAGGAAAATGGTAGAGTCAAACAATGTGTTGGTACTGTTACTTTGGATCAGATTAAAGACCCTCTCGACATCACTTGTAATGATGACGATGACAAAAGAGGTGGAAGATTGTGCTAC TGTAGGCGAAGATTCTGTGTTTGCGTAGGAAGGTAA
